# Prescribing of antipsychotics for people diagnosed with severe mental illness in UK primary care 2000–2019: 20-year investigation of who receives treatment, with which agents and at what doses

**DOI:** 10.1192/bjp.2024.186

**Published:** 2025-05

**Authors:** Alvin Richards-Belle, Naomi Launders, Sarah Hardoon, Kenneth K.C. Man, Elvira Bramon, David P.J. Osborn, Joseph F. Hayes

**Affiliations:** Division of Psychiatry, University College London, London, UK; Research Department of Practice and Policy, School of Pharmacy, University College London, London, UK; Centre for Medicines Optimisation Research and Education, University College London Hospitals NHS Foundation Trust, London, UK; Department of Pharmacology and Pharmacy, Li Ka Shing Faculty of Medicine, University of Hong Kong, Hong Kong; Laboratory of Data Discovery for Health (D24H), Hong Kong Science Park, Hong Kong; Camden and Islington NHS Foundation Trust, London, UK

**Keywords:** Antipsychotics, epidemiology, primary care, psychopharmacology, general adult psychiatry

## Abstract

**Background:**

Contemporary data relating to antipsychotic prescribing in UK primary care for patients diagnosed with severe mental illness (SMI) are lacking.

**Aims:**

To describe contemporary patterns of antipsychotic prescribing in UK primary care for patients diagnosed with SMI.

**Method:**

Cohort study of patients with an SMI diagnosis (i.e. schizophrenia, bipolar disorder, other non-organic psychoses) first recorded in primary care between 2000 and 2017 derived from Clinical Practice Research Datalink. Patients were considered exposed to antipsychotics if prescribed at least one antipsychotic in primary care between 2000 and 2019. We compared characteristics of patients prescribed and not prescribed antipsychotics; calculated annual prevalence rates for antipsychotic prescribing; and computed average daily antipsychotic doses stratified by patient characteristics.

**Results:**

Of 309 378 patients first diagnosed with an SMI in primary care between 2000 and 2017, 212,618 (68.7%) were prescribed an antipsychotic between 2000 and 2019. Antipsychotic prescribing prevalence was 426 (95% CI, 420–433) per 1000 patients in the year 2000, reaching a peak of 550 (547–553) in 2016, decreasing to 470 (468–473) in 2019. The proportion prescribed antipsychotics was higher among patients diagnosed with schizophrenia (81.0%) than with bipolar disorder (64.6%) and other non-organic psychoses (65.7%). Olanzapine, quetiapine, risperidone and aripiprazole accounted for 78.8% of all antipsychotic prescriptions. Higher mean olanzapine equivalent total daily doses were prescribed to patients with the following characteristics: schizophrenia diagnosis, ethnic minority status, male gender, younger age and greater relative deprivation.

**Conclusions:**

Antipsychotic prescribing is dominated by olanzapine, quetiapine, risperidone and aripiprazole. We identified potential disparities in both the receipt and prescribed doses of antipsychotics across subgroups. To inform efforts to optimise prescribing and ensure equity of care, further research is needed to understand why certain groups are prescribed higher doses and are more likely to be treated with long-acting injectable antipsychotics compared with others.

Antipsychotic medications are primarily indicated for the management of psychotic symptoms associated with severe mental illness (SMI), such as schizophrenia and bipolar disorder, and were prescribed to 756 000 people in England alone in 2019/20 (an almost 15% increase from 2015/16).^1^ Among individuals diagnosed with schizophrenia-spectrum disorders, antipsychotic use (versus non-use) is associated with a significantly lower long-term mortality rate.^2^ Despite this overall benefit, antipsychotic agents vary in their propensity for adverse reactions – with cardiometabolic effects, such as weight gain, dyslipidaemia and hyperglycaemia, major concerns of ‘second-generation’ antipsychotics, such as olanzapine, quetiapine and risperidone.^3^ Guidelines for schizophrenia management tend not to recommend individual antipsychotics,^4^ whereas guidelines for bipolar disorder are more specific and recommend olanzapine and quetiapine, in particular, across a range of presentations.^5^

In the UK, primary care services are responsible for the long-term prescribing of antipsychotics to individuals diagnosed with SMI. Data relating to antipsychotic prescribing in primary care are therefore essential for monitoring trends and identifying priorities for quality improvement and research. However, contemporary data on the SMI population are limited. Most reports have focused on other diagnoses, such as dementia^6^ or personality disorders,^7^ or on all-cause prescribing in children and young people^8^ and adults.^9^

Earlier studies documented antipsychotic prescribing practice in primary care.^10,11^ Prah et al investigated trends in schizophrenia, 1998–2007,^10^ and Hayes et al investigated bipolar disorder, 1995–2009.^11^ Both (a) illustrated the shift from prescribing first- to second-generation antipsychotics, (b) highlighted olanzapine, risperidone and quetiapine as the most frequently prescribed antipsychotics and (c) documented increases in the proportion of time spent receiving antipsychotic treatment, particularly for women. A more recent study reported further increases in antipsychotic prescribing to individuals diagnosed with bipolar disorder (from 37% of people in 2001 to 45% by 2018), with quetiapine, olanzapine and aripiprazole now the most frequently prescribed.^12^ Whether prescribing for schizophrenia and other psychoses has followed these trends is unknown, particularly following the licensing of aripiprazole in 2004.

Several studies report potential disparities in aspects of antipsychotic prescribing in UK primary care. A 2006 study in one London borough compared the management of Black versus White people diagnosed with psychosis and reported that Black people had greater odds of being prescribed long-acting injectable antipsychotics.^13^ A study (2005–2015) of diverse psychiatric diagnoses identified that men were, on average, prescribed higher antipsychotic doses than women, but results were not stratified by SMI diagnosis.^14^ Further contemporary explorations of these and other potential disparities, including stratification by age and relative deprivation, are needed to inform efforts to achieve equity of care.

## Aim and objectives

To inform future quality improvement and research into the optimal prescribing of antipsychotics, the overall aim of this study was to describe contemporary (2000–2019) patterns of antipsychotic prescribing for people diagnosed with SMI in UK primary care. Specific objectives were:

- to compare the characteristics of people diagnosed with SMI prescribed and not prescribed antipsychotics in primary care;
- to describe the most frequently prescribed antipsychotics and how this may have changed over time; and
- to describe the average prescribed daily antipsychotic dose over the first year of prescribing and explore whether doses vary according to diagnosis, ethnicity, age, gender and relative deprivation.

## Method

### Study design and data source

We conducted a longitudinal cohort study, using data from Clinical Practice Research Datalink (CPRD), to investigate antipsychotic prescribing from 1 January 2000 to 31 December 2019 in a cohort of people first diagnosed with SMI in primary care between 1 January 2000 and 31 December 2017. The study design is summarised in Supplementary Fig. 1 available at https://doi.org/10.1192/bjp.2024.186.

CPRD encompasses two databases (Aurum^15^ and GOLD^16^) which, collectively, contain the de-identified primary care records of over 62 million (current and historic) people from participating National Health Service (NHS) primary care practices. Over 98% of the UK population are registered in primary care, and CPRD is broadly representative. CPRD contains coded information on consultations, prescriptions, observations and referrals. We used data from the May 2022 and April 2023 builds of Aurum and GOLD, respectively.

### Participants

The cohort comprised individuals actively registered in primary care between 2000 and 2019 identified as first receiving an SMI diagnosis in their primary care record between 2000 and 2017. Following the NHS Quality and Outcomes Framework, SMI diagnosis was defined as a recorded Read or EMIS® code indicating a diagnosis of schizophrenia, bipolar disorder or other non-organic psychosis. In line with previous research,^17,18^ the latter includes non-affective psychoses, such as psychotic episodes, schizoaffective disorders, delusional disorder and non-organic psychosis not otherwise specified, but not primary affective psychoses (e.g. psychotic depression) where long-term antipsychotic treatment is not standard practice (see the online repository for the code list,^19^ which was verified by a clinician (J.F.H.)). SMI diagnoses are typically made by psychiatrists in secondary care and subsequently communicated to primary care. The validity of SMI diagnoses recorded in primary care has been established.^20^

### Outcomes: antipsychotics

People were considered exposed to antipsychotics if prescribed at least one antipsychotic in primary care during the study period (2000–2019). Antipsychotics could be initiated by general practitioners or specialists (e.g. psychiatrists), but must have been issued through primary care (standard practice for longer-term community prescriptions in the UK).^21^ Unless otherwise specified, antipsychotic prescription could pre-date the recording of SMI diagnosis in primary care (provided it was within the study period), given that antipsychotics may be initiated before a specific SMI diagnosis is formulated and/or communicated to primary care. Although antipsychotic prescription could pre-date SMI diagnosis, the requirement for first-recorded SMI diagnosis between 2000 and 2017 allowed for each person to accrue at least up to 2 years follow-up post-diagnosis (assuming they remained alive and registered in primary care).

#### Prescriptions of antipsychotics (objectives 1 and 2)

Antipsychotic medications (current and withdrawn) were identified through review of national and international sources.^22,23^ Search strategies, based on generic and common brand names (Supplementary Table 1), were developed to identify relevant product codes in CPRD code dictionaries. These codes were then used to extract data from prescription records, including product name, ingredient, prescription issue date, strength, formulation, route of administration, quantity, duration and de-identified free-text containing dosing instructions. We included both oral and injectable antipsychotics, but did not include prochlorperazine, given that it is primarily used as an antiemetic.

#### Antipsychotic dose (objective 3)

We computed the total daily prescribed oral antipsychotic dose for each of (up to) the first 12 prescription dates for individuals initiating antipsychotics in the study period. We considered doses of all tablet (e.g. extended release, sublingual) and liquid, but not injectable, formulations. Free-text dosage instructions (e.g. ‘take five tablets per day’) were converted to numerical quantities using a text-mining algorithm implemented in the R package *doseminer*.^24^ To enable comparison across agents, calculated doses were converted to olanzapine equivalents according to the Defined Daily Dose (DDD) method^25^ using *chlorpromazineR*^26^ (cariprazine and droperidol were not reported in the DDD method,^25^ and equivalence formulae for these antipsychotics came from Leucht et al^27^ and Gardner et al^28^ respectively). In the case of multiple prescriptions issued on the same date, we considered up to three unique prescriptions of each antipsychotic prescribed on a given date (>3 unique prescriptions of one medication was considered potentially erroneous).

### Stratifying variables

We extracted additional variables from CPRD to characterise the cohort and for stratified analyses. These included: year of birth, gender, ethnicity, geographic region, relative deprivation, date of first SMI diagnosis, SMI diagnosis and prescriptions of antidepressants and mood stabilisers. Where a person had multiple ethnicity categories coded, the most frequently recorded was used, or the most recent, if frequencies were equal. For people registered in England, if ethnicity was not coded in CPRD, ethnicity data were sourced from linked Hospital Episode Statistics data, where available. Geographic region refers to the location of the primary care practice at which the person was registered – and included Northern Ireland, Scotland, Wales and nine regions across England (defined according to Office for National Statistics categories). Linked small area-level data were used for people registered in England to provide information on relative deprivation (quintile of the 2019 English Index of Multiple Deprivation), derived according to individuals’ residential postcode (or, if unavailable, the practice postcode as a proxy). Where a person had more than one SMI diagnosis recorded over time, the most recent diagnostic category was used, as we considered this more likely to be accurate given a more complete clinical history, retaining the first diagnosis date. We used binary indicators for prescriptions of antidepressants and mood stabilisers (defined according to British National Formulary [BNF] chapters 4.3 and 4.2.3,^22^ respectively) during the study period. Follow-up time was calculated as the amount of time in years that individuals were registered in primary care during the study period (accounting for end of registration, death or administrative censoring).

### Statistical analysis

All analyses were conducted in R (version 4.3.1) using RStudio, and code is available in the online repository (https://github.com/Alvin-RB/antipsychotics_descriptive_study_cprd). Descriptive statistics were used to characterise the cohort, stratified by antipsychotic exposure status (objective one). To describe antipsychotic prescribing trends (objective two), we first calculated the number of people prescribed each antipsychotic at least once – overall and separately for long-acting injectables, and reported data for antipsychotics prescribed to ≥50 people. We then calculated period prevalence rates for the prescribing of antipsychotics, overall and for each antipsychotic, standardised to 1000 individuals, for each year during 2000–2019. Within each year, the numerator was the number of people that received at least one prescription for the antipsychotic over a denominator of the number alive, diagnosed with SMI and remaining registered in primary care. Allowing for recording delays, diagnosis could be recorded up to 2 calendar years after prescription to be considered ‘diagnosed’ in the given year. Period prevalence rates for the top 15 most frequently prescribed antipsychotics were represented using line graphs, overall and stratified by SMI diagnosis. Line graphs were also used to visualise the mean total daily prescribed oral antipsychotic dose (with 95% confidence intervals) for up to the first 12 prescription dates, stratified by diagnosis, ethnicity, age, gender and quintile of the 2019 English Index of Multiple Deprivation (objective three). Among those prescribed an antipsychotic more than once, we focused on the first 12 prescription dates among individuals identified as new users of antipsychotics in the study period, to ensure comparable prescribing periods. Assuming an average prescription duration of 28–30 days, we anticipated that this would approximate people's first year of prescribing. Where the number of daily doses prescribed was missing for a given prescription (16.8%), it was imputed (Supplementary Table 2) (we also undertook an analysis without this imputation). For missing daily dose values, the previous dose was carried forward for the missing observation only if the dose at the subsequent time-point was the same.

## Results

### Objective 1: characteristics of individuals prescribed and not prescribed antipsychotics in primary care

From a total of 514 526 people ever receiving a SMI diagnosis in the CPRD database during the study period, 309 378 were identified as having an SMI diagnosis first recorded in their primary care record between 2000 and 2017. From these, 212 618 (68.7%) were prescribed an antipsychotic in primary care at least once between 2000 and 2019, whereas 96 760 (31.3%) were not (Table 1).

**Table 1:** Characteristics of people prescribed and not prescribed antipsychotics in primary care between 2000 and 2019

	Not prescribed antipsychotic, *N* = 96 760	Prescribed antipsychotic, *N* = 212 618
Demographics		
Gender, *n* (%)		
Female	45 521 (47.0%)	102 449 (48.2%)
Male	51 232 (53.0%)	110 158 (51.8%)
Unknown	7	11
Ethnicity, *n* (%)		
Asian	4443 (5.2%)	12 695 (6.7%)
Black	6199 (7.2%)	14 920 (7.9%)
Mixed	2048 (2.4%)	4629 (2.4%)
Other	1536 (1.8%)	3282 (1.7%)
White	71 337 (83.4%)	153 493 (81.2%)
Unknown	11 197	23 599
Geographic region, *n* (%)		
East Midlands	2053 (2.1%)	4051 (1.9%)
East of England	3901 (4.0%)	8374 (3.9%)
London	21 401 (22.1%)	43 706 (20.6%)
North East	2620 (2.7%)	5384 (2.5%)
North West	13 844 (14.3%)	35 906 (16.9%)
Northern Ireland	828 (0.9%)	3685 (1.7%)
Scotland	6378 (6.6%)	15 842 (7.5%)
South East	15 888 (16.4%)	33 762 (15.9%)
South West	10 738 (11.1%)	19 829 (9.3%)
Wales	5137 (5.3%)	11 782 (5.5%)
West Midlands	11 124 (11.5%)	24 781 (11.7%)
Yorkshire and The Humber	2848 (2.9%)	5516 (2.6%)
English 2019 IMD quintile, *n* (%)^a^		
1 (Least deprived)	10 698 (13.1%)	20 434 (11.6%)
2	12 871 (15.7%)	25 232 (14.3%)
3	15 629 (19.1%)	32 090 (18.2%)
4	20 147 (24.6%)	43 938 (25.0%)
5 (Most deprived)	22 500 (27.5%)	54 373 (30.9%)
Unknown	14 915	36 551
Time actively registered in study period (years), median (IQR)	3.4 (1.2, 9.0)	5.6 (2.0, 12.9)
Mental health		
SMI diagnosis, *n* (%)		
Bipolar disorder	38 413 (39.7%)	70 137 (33.0%)
Other non-organic psychoses	45 207 (46.7%)	86 611 (40.7%)
Schizophrenia	13 140 (13.6%)	55 870 (26.3%)
Age at first SMI diagnosis, median (IQR)	36 (25, 52)	37 (27, 53)
Age at first SMI diagnosis (category), *n* (%)		
<30	36 018 (37.2%)	68 413 (32.2%)
30–39	19 506 (20.2%)	46 368 (21.8%)
40–64	26 881 (27.8%)	63 867 (30.0%)
65+	14 355 (14.8%)	33 970 (16.0%)
Year of SMI diagnosis, median (IQR)	2008 (2004, 2012)	2008 (2004, 2012)
Prescribed a mood stabiliser, *n* (%)^b^	21 954 (22.7%)	67 241 (31.6%)
Prescribed an antidepressant, *n* (%)^b^	51 558 (53.3%)	147 574 (69.4%)
No mood stabiliser or antidepressant, *n* (%)^b^		
At least one antidepressant or mood stabiliser	57 306 (59.2%)	162 522 (76.4%)
No antidepressant/mood stabiliser	39 454 (40.8%)	50 096 (23.6%)
Time from SMI diagnosis to end of follow-up (years), median (IQR)	5.7 (2.2, 10.6)	6.8 (3.2, 11.8)
Antipsychotics		
Antipsychotic initiation time-period, *n* (%)^c^		
<2000	–	13 895 (6.5%)
2000–2009	–	94 067 (44.2%)
2010–2019	–	104 656 (49.2%)
Prescribed antipsychotic prior to SMI diagnosis date, *n* (%)	–	73 038 (34.4%)
Ever prescribed oral antipsychotic, *n* (%)	–	208 693 (98.2%)
Time from SMI diagnosis to first oral antipsychotic (days), median (IQR)	–	28 (−78, 651)
Age at first oral antipsychotic, median (IQR)	–	38 (28, 54)
Age at first oral antipsychotic category, *n* (%)		
<30	–	59 971 (28.2%)
30–49	–	49 187 (23.1%)
40–64	–	66 226 (31.1%)
65+	–	33 309 (15.7%)
Ever prescribed LAI antipsychotic, *n* (%)	–	17 976 (8.5%)
Time from SMI diagnosis to first LAI antipsychotic (years), median (IQR)	–	3.1 (0.4, 7.6)
Age at first LAI, median (IQR)	–	44 (32, 61)
Time from first to last antipsychotic (years), median (IQR)	–	3.5 (0.8, 8.5)

IMD, Index of Multiple Deprivation; SMI, severe mental illness; LAI, long-acting injectable; IQR, interquartile range.

a. Among people registered at primary care practices in England only.

b. During the study period 2000–2019.

c. Among the 96 760 individuals not prescribed an antipsychotic during the study period, 1450 (1.50%) were prescribed an antipsychotic prior to the year 2000.

People prescribed and not prescribed antipsychotics were broadly similar demographically (Supplementary Fig. 2), but some regional differences were observed – with greater proportions prescribed antipsychotics in the North West of England, Northern Ireland and Wales. The proportion prescribed antipsychotics was higher among individuals diagnosed with schizophrenia (81.0%) than with bipolar disorder (64.6%) or other non-organic psychoses (65.7%). Among those not prescribed antipsychotics, over a fifth (22.7%) were prescribed mood stabilisers and over half (53.3%) antidepressants in the study period, but these proportions were higher among those prescribed antipsychotics (31.6 and 69.4%, respectively). The median time registered in primary care during the study period was shorter among those not receiving antipsychotics (3.4 versus 5.6 years). Comparisons are stratified by SMI diagnosis in Supplementary Tables 3–5.

Among those prescribed antipsychotics, almost all (98.2%) received at least one oral prescription. The median time from SMI diagnosis to first oral antipsychotic prescription was 28 (interquartile range [IQR], −78 to 651) days and from first to most recent or last antipsychotic prescription was 3.5 (IQR, 0.8 to 8.5) years. Over a third (34.4%) were prescribed an antipsychotic with a median (IQR) of 16 (3 to 53) months prior to having an SMI diagnosis recorded in their primary care record. Of those prescribed an antipsychotic overall, 8.5% were prescribed a long-acting injectable, but this proportion ranged from 4.5 to 16.4% among those diagnosed with bipolar disorder and schizophrenia, respectively (Supplementary Tables 3–5). Stratified by ethnicity, the proportion prescribed a long-acting injectable was highest among Black people (9.2%), very similar among Asian and Mixed individuals (6.8 and 6.9%, respectively) and lowest among White people and those of other ethnicities (5.5 and 4.5%, respectively). Additional stratification by diagnosis with ethnicity and gender is shown in Supplementary Tables 6 and 7.

### Objective 2: antipsychotic prescribing trends

After excluding 1171 prescriptions (across 764 people) considered potentially erroneous duplicates, the 212 618 people diagnosed with SMI and prescribed an antipsychotic had a total of 11 745 996 prescriptions, covering 33 different medications, between 2000 and 2019. Olanzapine was prescribed at least once to 91 961 (43.3%) individuals and was the most frequently prescribed, followed by quetiapine (*n* = 70 250, 33.0%), risperidone (*n* = 63 893, 30.1%) and aripiprazole (*n* = 44 344, 20.9%) (Supplementary Fig. 3). These four antipsychotics accounted for 78.8% of all prescriptions. The most frequently prescribed first-generation antipsychotics were chlorpromazine (*n* = 17 195, 8.1%) and haloperidol (*n* = 17 119, 8.1%). Clozapine was infrequently prescribed in primary care (*n* = 5346, 2.5%). Trends were similar when considering first- and second-line medications (Supplementary Table 8). The most frequently prescribed long-acting injectables were flupentixol and zuclopenthixol (Supplementary Fig. 4).

**Fig. 1:**
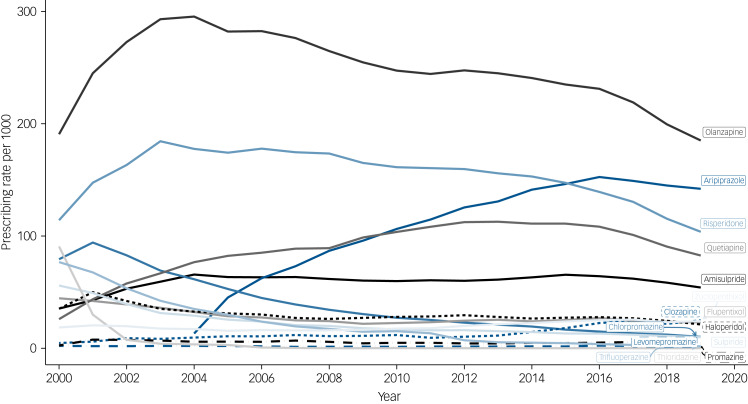
Annual prevalence rates for the prescribing of antipsychotics to people diagnosed with schizophrenia.

**Fig. 2:**
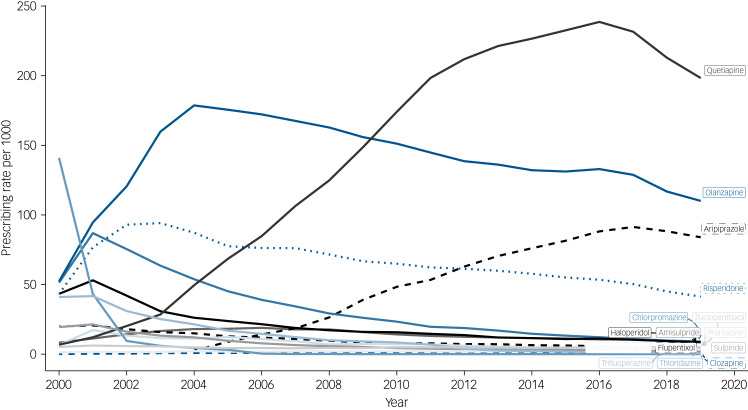
Annual prevalence rates for the prescribing of antipsychotics to people diagnosed with bipolar disorder.

The overall prevalence of antipsychotic prescribing was 426 (95% CI, 420 to 433) per 1000 people in the year 2000, reaching a peak of 550 (95% CI, 547 to 553) in 2016, then decreasing to 470 (95% CI, 468 to 473) in 2019 (Supplementary Fig. 5). Annual prevalence rates for individual antipsychotics varied over time (Supplementary Fig. 6) and according to SMI diagnosis. Among individuals with a diagnosis of schizophrenia, olanzapine was most frequently prescribed, and, for most of the time-period, this was followed by risperidone (Fig. 1). However, in 2015, aripiprazole overtook risperidone. Among those with a diagnosis of bipolar disorder, olanzapine had been the most frequently prescribed up until to 2009, after which it was overtaken by quetiapine (Fig. 2). Among people diagnosed with other non-organic psychoses, prescribing prevalences for quetiapine, aripiprazole and risperidone were all relatively similar after 2016, but olanzapine was the most frequently prescribed throughout (Supplementary Fig. 7).

### Objective 3: variation in average prescribed daily antipsychotic dose over people's first year of prescribing

Of the 212 618 people prescribed antipsychotics between 2000 and 2019, 194,979 were identified as newly prescribed an oral antipsychotic in primary care during the study period. After exclusions (15 703 for receiving just one prescription and 42 because of having no known doses across their first 12 prescription dates), a total of 179 234 individuals, with 1 780 077 prescription dates, were included.

**Fig. 3:**
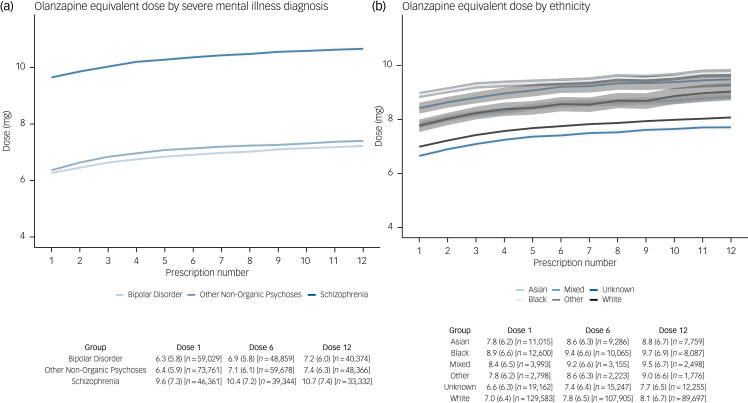
Mean total daily prescribed oral antipsychotic dose over the first 12 prescriptions – stratified by (a) severe mental illness diagnosis and (b) ethnicity. Graphs show the mean olanzapine equivalent dose (mg) at each time-point, with 95% confidence intervals. The table beneath the graph shows the corresponding mean (s.d.) olanzapine equivalent doses at prescription date 1, 6 and 12, with the number of observations at each time-point. The overall median time between prescription dates was 28 days.

Mean total daily prescribed oral antipsychotic doses varied across subgroups, but all tended to increase slightly over the first 12 prescription dates. Stratified by SMI diagnosis, individuals diagnosed with schizophrenia were prescribed the highest doses (mean [s.d.] daily dose at 12th prescription date: 10.7 [7.4] mg olanzapine equivalent dose), whereas those diagnosed with bipolar disorder were prescribed the lowest doses (7.2 [6.0] mg) (Fig. 3(a)). When stratified by ethnicity, Black individuals were prescribed the highest doses (9.7 [6.9] mg), followed by Mixed (9.5 [6.7] mg), Other (9.0 [6.6] mg), then Asian (8.8 [6.7] mg), whereas White people were prescribed the lowest doses (8.1 [6.7] mg) (Fig. 3(b)). Mean daily doses were higher in males compared with females (Supplementary Fig. 8), in younger compared with older (65+) people (Supplementary Fig. 9) and in people in the more versus less deprived quintile of the English Index of Multiple Deprivation (Supplementary Fig. 10). Trends were similar in analyses that did not impute missing number of daily doses (not shown).

## Discussion

Using a large, longitudinal sample of 309 378 people diagnosed with SMI between 2000 and 2017, we provide contemporary data (2000–2019) on antipsychotic prescribing practice in UK primary care. We identify several important findings relevant to informing future quality improvement and research into optimal prescribing, including the following: (a) prescribing is dominated by olanzapine, quetiapine, risperidone and aripiprazole – accounting for 79% of all prescriptions; (b) potential disparities in prescribed antipsychotic and dose exist – namely higher doses prescribed to people with characteristics such as ethnic minority status and greater relative deprivation and (c) almost a third of individuals with a contemporaneous SMI diagnosis are not prescribed antipsychotics in primary care.

Overall, olanzapine was the most frequently prescribed antipsychotic throughout the study period. Stratified by diagnosis, this remained true for schizophrenia and other non-organic psychoses, but not for bipolar disorder, for which, since 2010, quetiapine was most frequently prescribed. Adverse cardiometabolic effects are a major concern of second-generation antipsychotics, and when antipsychotics are ranked according to their impact on cardiometabolic parameters, olanzapine is consistently identified as one of the worst-ranking, particularly for changes in body weight, body mass index and low-density lipoprotein cholesterol.^3^ The continued popularity of olanzapine may be due to a perceived greater efficacy compared with other antipsychotics,^29^ despite most antipsychotics being considered broadly comparable in efficacy.^30^ Alternatively, for individuals well established on olanzapine, it may be due to the perceived relapse risk presented by switching to a different antipsychotic with less cardiometabolic burden.

The 2004 licensing of aripiprazole led to a major change in prescribing, whereby prescriptions of aripiprazole have increased year on year – now making aripiprazole one of the most frequently prescribed antipsychotics. This is an important development as current evidence suggests that aripiprazole is associated with fewer adverse cardiometabolic effects, especially when compared with olanzapine and quetiapine.^3,31^ However, some reviews have reported aripiprazole to be less efficacious than some other antipsychotics, such as olanzapine and risperidone,^29,32^ although others report no differences.^30^ Aripiprazole is also suggested to exacerbate psychotic symptoms among individuals with significant prior antipsychotic exposure.^33^ Aripiprazole is still one of the most recently licensed antipsychotics, and current popularity might reflect effectiveness of pharmaceutical marketing or a novelty effect in the face of limited innovations in the development of new antipsychotics. These issues highlight the difficulty, but necessity, of evaluating the risk/benefit ratio of individual antipsychotics.

Influential UK guidelines such as those from the National Institute for Health and Care Excellence (NICE) will have impacted prescribing during the study period. However, although early NICE guidelines for schizophrenia (2002) recommended specific antipsychotics (i.e. amisulpride, olanzapine, quetiapine, risperidone, zotepine),^34^ subsequent revisions for the management of psychosis and schizophrenia (2009 and 2014) are non-specific with regards to individual antipsychotics (except for clozapine as a third-line treatment), possibly permitting greater flexibility for patients and clinicians.^35^ In contrast, NICE guidelines for bipolar disorder have greater specificity. In 2006, olanzapine was the only antipsychotic recommended for long-term management, with olanzapine, quetiapine and risperidone recommended for acute mania, and quetiapine for acute depressive symptoms.^36^ This list is expanded in the 2014 guidelines – with asenapine, aripiprazole, olanzapine, quetiapine and risperidone now recommended for long-term management; haloperidol, olanzapine, quetiapine and risperidone for acute mania/hypomania; and olanzapine (with fluoxetine) and quetiapine for acute depressive symptoms.^5^ Quetiapine's broad recommendations likely makes it a more versatile and thus popular choice; however, we observed that it was already the most frequently prescribed antipsychotic in bipolar disorder prior to 2014.

If it were possible to optimise current prescribing, then efforts focusing on olanzapine, quetiapine, risperidone and aripiprazole could have a large impact on the SMI population, given their very widespread use (79% of all antipsychotic prescriptions). Studies of the comparative safety and effectiveness of aripiprazole are particularly warranted, given aripiprazole's potential to reduce cardiometabolic risk alongside concerns of possibly lesser effectiveness. Conversely, some antipsychotics are rarely prescribed, and so there is limited opportunity to learn about their relative risks and benefits in pharmacoepidemiologic studies using routine clinical data.

To inform future quality improvement and research, we sought to describe current practice and identify subgroups that may potentially be at more risk of dose-dependent adverse reactions. We found that, on average, higher doses were prescribed to individuals with the following characteristics: diagnosis of schizophrenia, ethnic minority status, male gender, younger age and greater relative deprivation. In addition, we replicated greater use of long-acting injectables among Black individuals.^13^ To our knowledge, this is the first study to report potential disparities according to ethnicity and relative deprivation in prescribed antipsychotic dose in UK primary care. For ethnicity, people from all ethnic minorities were prescribed higher doses than White people. Confidence intervals for ethnic minority groups were inevitably wider than, but never overlapped with, the White group – owing to smaller sample sizes reflecting minority status. We did not aim to estimate whether certain characteristics are causally related to being prescribed higher doses or to identify potential mediating factors, and therefore our analyses were unadjusted, as recommended for descriptive studies.^37^ Clearly, multiple factors may influence decisions to prescribe at a certain dose, and further research is needed to disentangle the effects of these factors in order to explain, and potentially inform efforts to reduce, these potential disparities. Causal inference approaches accounting for a wide range of potential confounders (e.g. markers of severity, access to care), alongside qualitative approaches examining clinical decision-making, would be informative. Moreover, these data might prompt local services to audit their prescribing and, if differences are found, to delve into the specific reasons and processes leading to such potential disparities.

Finally, there was a trend of declining antipsychotic prescribing rates over the later study years and, overall, almost one-third of people with a contemporaneous SMI diagnosis were not prescribed antipsychotics in primary care (but this proportion varied across diagnoses – ranging from 19 to 35% in individuals diagnosed with schizophrenia and bipolar disorder, respectively). This is potentially concerning, given reports of worse outcomes, including higher mortality, among individuals diagnosed with schizophrenia-spectrum disorders not prescribed antipsychotics.^2^ Noting that fewer than 2% of these people were identified as prescribed an antipsychotic in primary care prior to the study period, it is difficult to ascertain if the remainder were truly unexposed based on primary care records alone. Although the shorter follow-up time reduced the opportunity to identify antipsychotic prescriptions, this group still had a median follow-up of 3.4 years – seemingly sufficient to detect regular prescribing. Nevertheless, a small proportion will likely have been prescribed antipsychotics exclusively in secondary care (e.g. as inpatients) – an issue particularly relevant for clozapine. Alternative explanations might include: individuals declining antipsychotics and/or instead receiving psychological interventions or non-antipsychotic pharmacotherapies (particularly for those diagnosed with bipolar disorder); people with brief or less severe psychotic episodes; or perhaps some were not in contact with services following diagnosis (although many were prescribed other psychiatric medications).

### Strengths and limitations

A strength of this study is the large longitudinal cohort of people diagnosed with SMI, derived from CPRD – which is broadly representative of the UK population.^15,16^ CPRD includes all prescriptions issued in primary care and is therefore accurate in terms of planned treatment, and prescriptions issued repeatedly suggest adherence to that medication. We covered a 20-year period, enabling the identification of contemporary trends in prescribing for SMI, whereas other studies focused on other diagnoses or on all-cause prescribing. When analysing antipsychotic dose, it was important to consider dose over multiple time-points in order to capture potential changes, rather than just the starting dose, which may not have accurately reflected ongoing management.

This study also has limitations. First, we included prescriptions issued only from primary care. The 2014 National Audit of Schizophrenia reported that 20% of people under the care of community mental health teams were prescribed clozapine,^38^ whereas fewer than 2% of people diagnosed with schizophrenia in our cohort had a prescription for clozapine in primary care. We were therefore not able to comment in detail on clozapine prescribing due to likely limited coverage. We also did not have data on dispensing or individual patient adherence (although repeat prescriptions issued with a regular cadence suggest adherence). Studies combining prescribing and dispensing data across primary and secondary care are needed to characterise the complete national picture on antipsychotic prescribing; such studies might soon be feasible with the continued development of national data resources.^39^ Second, there were some missing data in some key variables, including the number of daily doses (which we imputed) and ethnicity (which we considered as a separate category in dose analyses). Moreover, we studied broad ethnic groups, consistent with UK-census high-level ethnicity categories, and focused on between-group, rather than within-group, heterogeneity. Studies of more specific ethnic groups are needed, but will be challenging because of smaller sample sizes, missing data and greater misclassification risk. Addtionally, although ethnicity should be self-reported in primary care, we cannot verify this assumption. Third, the study period went up to 2019 and therefore did not cover the COVID-19 pandemic period. Initial evidence from an England-wide analysis suggests that antipsychotic prescribing remained relatively stable in the SMI population during the pandemic period,^40^ but studies with a greater SMI focus are warranted. Finally, 8% of those considered exposed to antipsychotics received just one prescription during the study period; alternative exposure definitions (e.g. at least two prescriptions) might have yielded slightly different results.

## Supporting information

Richards-Belle et al. supplementary material Richards-Belle et al. supplementary material

## Data Availability

Data underlying this study were accessed via CPRD under approved protocol no. 21_000729. Authors are not able to share the data directly; however, data can be accessed directly from CPRD following approval and licensing (see https://cprd.com/ for further details). The analytic code and materials supporting the findings are available in the online repository (https://github.com/Alvin-RB/antipsychotics_descriptive_study_cprd).
